# Bone Health in Ajman: A Cross‐Sectional Study of Bone Mineral Density Among Adults

**DOI:** 10.1155/joos/8992219

**Published:** 2026-02-22

**Authors:** Nermeen Mohammed Abdelmonen Mohammed, Munira Zainuddin Ali, Khadija Jalal, Athira Suresh Kumar, Sathya Subramoniam Iyer, Farhana Fathin, Fatima Shamim, Raseena Aboobacker Abdulla, Nafeesa Abdul Kareem, Arundeep Arora, Jayadevan Sreedharan, Jayakumary Muttappallymyalil

**Affiliations:** ^1^ Center of Imaging, Thumbay University Hospital, Ajman, UAE; ^2^ College of Medicine, Gulf Medical University, Ajman, UAE, gmu.ac.ae

**Keywords:** bone mineral density, DEXA, menopause, *T* score

## Abstract

**Background:**

Bone mineral density (BMD) is a quantitative measurement of the amount of inorganic minerals which are present in a particular volume of bone. Variations in BMD are dependent on a variety of factors including age, sex, race, family history, age at menarche and menopause, and the presence of any other health conditions. This study aims to determine the association between categories of BMD among individuals referred to the department of radiodiagnosis for dual‐energy x‐ray absorptiometry (DEXA) scan and associated clinico‐epidemiological factors.

**Methods:**

A record‐based cross‐sectional study was used to collect the data of 335 individuals who presented to the department of radiodiagnosis at Thumbay University Hospital, Ajman, UAE., for DEXA scan from 2015 to 2022. SPSS Version 28 was used for analysis. Inferential statistics like chi‐square was performed to establish the association between variables, and a *p*‐value less than or equal to 0.05 was taken to be statistically significant.

**Results:**

Among 335 individuals, 64.8% were females and 35.2% were males. Majority of the participants belonged to the 40–59 age group at 54.3%, were of African origin at 53.7%, and were classified as overweight/obese at 86.3%. The left hip *T* score was statistically significant with geographical origin (*p* < 0.001) and menopausal status (*p* < 0.01) as well as a history of disc disorders (*p* = 0.05). The spine *T* score was statistically significant in terms of its association with age (*p* = 0.04), geographical origin (*p* < 0.001), and menopausal status (*p* < 0.001). The combination *T* score taken based on both spine and hip *T* scores was found to be statistically significant in terms of association with sex (*p* = 0.05), age (*p* < 0.001), geographical origin (*p* < 0.001), and body mass index (*p* = 0.003).

**Conclusion:**

Our study, aimed at determining the association between different categories of BMD and clinicoepidemiological factors of the participants, found that increasing age, underweight/normal weight category, being male, and belonging to the African region were significantly associated with the combination *T* scores.

## 1. Background

Bone mineral density (BMD) [1] is a quantitative measurement of the amount of inorganic minerals like calcium and phosphorus, which are present in a particular volume of the bone. Variations in the BMD [2, 3] of an individual are dependent on a variety of nonmodifiable factors like age, sex, ancestry, family history, age at menarche and menopause, and the presence of any other health conditions. Modifiable factors such as the level of physical activity, weight, and lifestyle factors which include practices like smoking, excessive alcohol intake, serum calcium, and vitamin D levels also influence the BMD of an individual. The current gold standard imaging test for the measurement of BMD and the diagnosis of associated disorders is the dual‐ energy X ray absorptimetry (DEXA) or DEXA scan [4]. The most prevalent disorder associated with a variation in BMD is osteoporosis, the most common metabolic bone disorder in the world [5]. Osteoporosis is a condition characterized by a decrease in the BMD of an individual resulting in a deterioration of the bone strength, making individuals more susceptible to fractures. Osteoporosis [6] is preceded by a stage called osteopenia, where there is a decrease in the BMD of the individual, which is below the normal reference value, but not low enough to be diagnosed as osteoporosis. For a diagnosis of osteopenia, the *T* score value from the DEXA scan must fall between −1 and −2.5. A value lesser than −2.5 meets the diagnostic criteria for osteoporosis [5].

Recently, a study [7] conducted among a Saudi Arabian sample population concluded that the prevalence of osteoporosis had increased significantly when compared to earlier reports, where 63.6% of the men and 52.8% of the females were diagnosed with osteoporosis. Also, males who were vitamin D deficient showed osteoporosis. The research of Taei et al. [8] showed that 53.2% of the patients had osteopenia, 27.1% had osteoporosis, and 0.3%, severe osteoporosis. Findings from an Indian study [9] indicated that 30.5% and 44.2% were suffering from osteoporosis and osteopenia, respectively. This study concluded that significant risk factors for the development of osteopenia and osteoporosis included high systolic blood pressure, high triglyceride levels, poor sleep quality, and high levels of C‐reactive protein. As reported in a study [10] conducted in the Gansu province, the prevalence of osteopenia and osteoporosis was, respectively, 27.09% and 9.65%. Among the elderly men, 26.68% had osteopenia and 8.08% were seen to have osteoporosis. In postmenopausal women, the factors that were seen to be significantly associated with a risk of developing osteoporosis were age, age at menopause, duration of menopause, body mass index (BMI), level of education, and alcohol consumption. In elderly men, age, BMD, smoking status, alcohol consumption, physical activity, and sun exposure were seen to be significantly associated with a risk of developing osteoporosis. Shi et al. [11] reported that those with osteoporosis and osteopenia were more likely to have low serum levels of minerals like calcium and vitamin D and a higher chance of having diabetes, high blood pressure, and a history of cardiovascular diseases.

A decrease in BMD represents a major public health problem. Due to better healthcare services in UAE [12], there is a larger population of senior citizens in UAE, increasing the prevalence of conditions such as osteoporosis. Vitamin D deficiency is one of the main attributing factors to the development of BMD disorders such as osteoporosis, osteopenia, and osteomalacia [13]. The results of a study [14] conducted among the Emirati population showed that 72% of the participants were either vitamin D deficient or insufficient. Obesity has also been shown to contribute to developing low BMD (LBMD) [15]. UAE is ranked 26th in the Global Obesity Observatory rankings [16] for obesity among male adults and 20th for female adults.

Healthcare professionals need to know about the prevalence of conditions that affect BMD such as osteoporosis and its association with age, sex, BMI, cardiovascular diseases, vitamin deficiencies, and other factors. This allows them to be sufficiently equipped to advise patients on the various preventive measures that can be taken to prevent the incidence of such conditions. Policymakers can use the research as a guide to identify the areas in which more focus is required so that strong policies can be passed that protect the general population from undesirable health outcomes and, at the same time, elevate the quality of care. Researchers can think of conducting case–control or cohort studies to ascertain the factors associated with the level of BMD.

The present research can create awareness among the general public regarding the factors that lead to osteoporosis and how it can be prevented. Moreover, the lack of recent literature on BMD levels among adults in the UAE highlights the need for this research. This study aims to determine the association between the category of BMD‐associated clinical‐epidemiological factors among individuals referred to the department of radiodiagnosis for DEXA scan.

## 2. Materials and Methods

This study included all adults who underwent DEXA scans in the department of radiodiagnosis at Thumbay University Hospital, Ajman, U.A.E., from the years 2020 to 2022. Records with incomplete data were excluded.

After obtaining approval from the Institutional Review Board of Gulf Medical University (Ref. no. IRB‐COM‐STD‐78‐May‐2023), permission to access the medical records was obtained through the Director of Thumbay University Hospital. Data collection of the records consisted of sociodemographic details such as age, sex, geographical origin, height, and weight. BMI was calculated from the height and weight recorded while performing the DEXA scan. The level of BMI was categorized based on the formula: weight (kg)/[height (m)]^2^.

The standard categorization of BMI by the CDC indicates less than 18.5 as underweight, 18.5–24.9 as normal, 25.0–29.9 as overweight, and 30.0 and above as obese. DEXA is the most accurate way to measure BMD. BMD was performed at the left hip and the lumbar spine (L2 to L4) using a DEXA densitometer. WHO criteria were used for categorizing the respondents based on DEXA results.

A structured proforma was developed to extract relevant data from the records in alignment with the study objectives. The face and content validity was checked by two experts from the department of radiodiagnosis. The suggestions obtained from the experts were incorporated into the proforma and sent back to the experts for final approval. A pilot study was carried out using five records to assess the feasibility, and the time taken to fill the proforma before finalizing. The proforma was prepared on an excel spreadsheet, and the data were entered on it. The SPSS Version 28 was used for analysis. Descriptive and inferential statistical methods were used to analyze the data. The association between the level of BMD and factors was analyzed using the chi‐square test. A *p* value of less than 0.05 was considered to be statistically significant. All missing data were eliminated from the study.

The complete dataset used for analysis is available in the Supporting Information (Supporting File 1).

## 3. Results

This study includes 335 participants, most of whom are 40–59 years old (182; 54.3%). The distribution of participants according to sex was 217 (64.8%) females and 118 (35.2%) males. The geographical origins were classified according to WHO regions: Africa, Eastern Mediterranean, and other regions (Region of Americas, South‐East Asia, Europe, and Western Pacific). Over half of the participants belonged to the African region (180; 53.7%), and 35.8% belonged to the Eastern Mediterranean region. The majority of the participants (289; 86.3%) were classified as either overweight or obese according to the BMI classification, and 13.7% belonged to the category of underweight/normal. According to marital status, 322 (96.1%) participants were married. The majority of the female participants (159; 73.3%) were observed to have attained menopause, whereas 26.7% were still in the active reproductive phase. Hyperlipidemia and hypertension were observed to be the most common chronic conditions, with a prevalence of 45.7% (153) and 43.9% (147), respectively. Diabetes affected 29.9% (100) of the participants. Over half of the participants (79; 62.7%) out of 126 participants tested were observed to be deficient in Vitamin D.

Concerning their history of musculoskeletal disorders, 52.8% of the individuals did not have any history of MSK disorders, 15.8% had a past diagnosis of osteoporosis confirmed by DEXA and had come for a follow‐up DEXA scan, and 11.6% of them had other MSK disorders like gout, osteomalacia, scoliosis, carpal tunnel syndrome, rotator cuff tear, and others.

LBMD is found in those with either osteopenia or osteoporosis based on *T* score. The combination *T* score was taken based on the left hip and spine *T* scores; patients with LBMD based on either hip or spine *T* score were considered to have LBMD in the combination *T* score. As shown in Table 1, 127 participants (37.9%) and 158 participants (47.2%) had LBMD based on spine *T* score and left hip *T* score alone, respectively. Based on the combination score, 166 (49.6%) had LBMD.

**Table 1:** Classification of participants into different categories based on spine and left hip *T* score.

Categories	Spine *T* score		Left hip *T* score		Combination	
	No.	%	No.	%	No.	%
Normal bone mineral density	208	62.1	177	52.8	169	50.4
Low bone mineral density	127	37.9	158	47.2	166	49.6

Additional details of the dataset are available in the Supporting Information (Supporting File 1).

Table 2 shows the association between left hip *T* score and sociodemographic characteristics. Abnormal scores are those who are categorized under osteopenia and osteoporosis. A statistically significant association using the chi‐square analysis was seen between left hip *T* score and geographical origin (*p* ≤ 0.001). Individuals from African region, 115 (63.9%) had a normal score. On the other hand, 72 (60%) of the Eastern Mediterranean region and 21 (60%) of the other regions had an abnormal score. A statistically significant association using the chi‐square analysis was also observed between left hip *T* score and menopausal status (*p* = 0.003). Out of those who attained menopause, a higher fraction of the females (52.2%) were observed to have an abnormal left hip *T* score.

**Table 2:** Association between left hip *T* score and sociodemographic features.

Sociodemographic features	Group	Left hip *T* score				*p* value
		Normal		Abnormal		
		No.	%	No.	%	
Sex	Female	117	53.9	100	46.1	NS
	Male	60	50.8	58	49.2	

Age	22–39	13	48.1	14	51.9	NS
	40–59	98	53.8	84	46.2	
	> 60	66	52.4	60	47.6	

Geographical origin	African region	115	63.9	66	36.1	< 0.001
	EMR	48	40	72	60	
	Others	14	40	21	60	

BMI	Underweight/normal	24	52.2	22	47.8	NS
	Overweight/obese	153	52.9	136	47.1	

Menopause	Yes	76	47.8	83	52.2	< 0.01
	No	41	70.7	17	29.3	

Vitamin D deficiency status	Yes	31	39.2	48	60.8	NS
	No	16	34	31	66	

Abbreviation: EMR, Eastern Mediterranean region.

Among the participants with a history of spondylosis, most (58.3%) had abnormal left hip *T* scores compared to the 41.7% who had normal *T* scores. However, this was not found to be statistically significant. Chi‐square analysis found a statistically significant association between history of disc disorders and left hip *T* score (*p* = 0.05), with a majority of participants (65.2%) with disc disorders having an abnormal left hip *T* score (Table 3).

**Table 3:** Association between left hip *T* score and other factors.

History of MSK disorders	Group	Left hip *t* score				*p* value
		Normal		Abnormal		
		No.	%	No.	%	
Disc disorders	Yes	8	34.8	15	65.2	0.05
	No	169	54.2	143	45.8	

Osteoarthritis/rheumatoid arthritis	Yes	13	41.9	18	58.1	NS
	No	164	53.9	140	46.1	

Spondylosis	Yes	5	41.7	7	58.3	NS
	No	172	53.3	151	46.7	

Other MSK disorders	Yes	20	51.3	19	48.7	NS
	No	157	53	139	47	

A statistically significant association determined through chi‐square analysis was found between spine *T* score and age (*p* = 0.04). Most participants (44.4%) with abnormal spine *T* score were found to belong to the 22–39 age category. Among the age groups of 40–59 and > 60, most had normal spine *T* scores at 57.1% and 70.6%, respectively. A statistically significant association was seen between spine *T* score and geographical origin (*p* ≤ 0.001). Most of those from the Eastern Mediterranean region (63, 52.5%) and other regions (19, 54.3%) had an abnormal score. A statistically significant association was also seen between spine *T* score and menopausal status (*p* ≤ 0.001). Among the patients who had attained menopause, 46.8% had abnormal *T* scores, while among premenopausal women, only 17.2% had abnormal *T* scores (Table 4).

**Table 4:** Association between spine T score and sociodemographic features.

Sociodemographic features	Group	Spine *T* score				*p* value
		Normal		Abnormal		
		No.	%	No.	%	
Sex	Female	131	60.4	86	39.6	NS
	Male	77	65.3	41	34.7	

Age	22–39	15	55.6	12	44.4	0.04
	40–59	104	57.1	78	42.9	
	> 60	89	70.6	37	29.4	

Geographical origin	African region	135	75	45	25	< 0.001
	Eastern Mediterranean region	57	47.5	63	52.5	
	Others	16	45.7	19	54.3	

BMI	Underweight/normal	30	65.2	16	34.8	NS
	Overweight/obese	178	61.6	111	38.4	

Menopause	Yes	83	52.2	76	46.8	< 0.001
	No	48	82.8	10	17.2	

Vitamin D deficiency status	Yes	38	48.1	41	51.9	NS
	No	23	48.9	24	51.1	

Chi‐square analysis revealed a statistically significant association between the combination of *T* score and sex (*p* = 0.05). Among males, 56.8% had an abnormal combination *T* score. A statistically significant association was also observed between the combination of *T* score and age (*p* ≤ 0.001), with participants aged > 60 years showing the highest prevalence of abnormal scores (63.5%), compared to 48.1% and 40.1% in the 40–59 and 22–39 age groups, respectively. Similarly, a statistically significant association was also found between the combination of *T* score and geographical origin (*p* ≤ 0.001); 58.9% (106) of participants from the African region had an abnormal combination *T* score. In addition, *T* score was significantly associated with BMI (*p* = 0.003), with 69.6% (32) of participants classified as underweight or normal exhibiting abnormal scores (Table 5).

**Table 5:** Association between combination T score and sociodemographic features.

Sociodemographic features	Group	Combination *T* score				*p* value
		Normal		Abnormal		
		No.	%	No.	%	
Sex	Female	118	54.4	99	45.6	0.05
	Male	51	43.2	67	56.8	

Age	22–39	14	51.9	13	48.1	< 0.001
	40–59	109	59.9	73	40.1	
	> 60	46	36.5	80	63.5	

Geographical origin	African region	74	41.1	106	58.9	< 0.001
	Eastern Mediterranean region	71	59.2	49	40.8	
	Others	24	68.6	11	31.4	

BMI	Underweight/normal	14	30.4	32	69.6	0.003
	Overweight/obese	155	53.6	134	46.4	

Menopause	Yes	89	56	70	44	NS
	No	29	50	29	50	

Vitamin D deficiency status	Yes	48	60.8	31	39.2	NS
	No	30	63.8	17	36.2	

## 4. Discussion

Concerning sex, no association was found with spine *T* score, with similar distribution seen among all categories. Other influencing factors such as lifestyle and diet among the participants may have contributed to the result, as well as the higher proportion of females among the study population. However, a retrospective study done in India [17] has demonstrated the decline in BMD to be greater among women as compared to men. Another study [18] found that age‐related decline in BMD is more pronounced in women, compared to men, and the different factors which influence this are variable in different age groups.

Participants in the current study from other regions (Region of Americas, South‐East Asia, Europe, and Western Pacific regions) and Eastern Mediterranean region had a little over half (19; 54.3%) of their respective population categorized as abnormal spine *T* score. The influence of factors such as exposure of vitamin D, sociocultural factors, and dietary differences among populations may explain this finding. A study [19] conducted in Saudi Arabia among Saudi older adults reported prevalence of osteoporosis and osteopenia in lumbar spine to be 11.8% and 41.2%, respectively.

The current study found a significant association between age and spine T‐scores, with a higher proportion of abnormal scores observed in the 22–39 age groups. Several studies have shown that factors such as physical activity levels [20] and lifestyle choices—including diet, smoking, and alcohol consumption [21]—can influence BMD during early adulthood. Regarding BMI, a slightly higher proportion of participants with underweight or normal BMI had abnormal spine *T*‐scores. These findings align with a previous study [22] conducted among older diabetic patients, which reported that higher BMI was associated with greater BMD in the lumbar spine.

Menopausal status and spine *T* score were shown to have significant association in the current study, with a higher proportion of postmenopausal women having abnormal *T* score as compared to premenopausal women. This is in line with the results of a study [23] done in Iraq. Another cross‐sectional study done among 342 women from Saudi Arabia also stated that menopause is associated with a reduction in the estrogen levels, which consequently has been associated with a higher rate of BMD disorders [24].

Estrogen, which is responsible for preventing bone loss, declines in postmenopausal women. This contributes to the LBMD seen in postmenopausal women [25]. The current study shows no significant association between vitamin D status and spine *T* score, with roughly uniform distribution of normal and abnormal *T* scores among deficient and nondeficient participants. This is in contrast to a study [26] performed with obese Saudi women where a significant association was found with 76.5% of those with osteopenia, having mild‐to‐moderate deficiency. Differences in the type of sample and the limited testing of vitamin D status of the participants may explain these results.

The current study shows that there is no significant association between sex and left hip *T*‐score, as indicated by the *p*‐value. Both females and males have almost similar proportions of normal and abnormal *T*‐scores. Similarly, age does not have a significant association with left hip *T*‐score. Across different age groups, the proportions of normal and abnormal *T*‐scores do not vary significantly. These similarities in proportions may be attributed to relative homogeneity of the study population in terms of the factors that influence bone health. These factors include lifestyle, diet, and physical activity. If there were no significant differences between males, females, and the age groups in these factors, it could contribute to the lack of association with *T* scores.

In contrast, geographical origin shows a significant association with left hip *T*‐score. Individuals from the Eastern Mediterranean region and other regions have a higher proportion of abnormal *T*‐scores compared to those from the African region. This may be due to factors such as higher prevalence of calcium and vitamin D deficiencies and limited sunlight exposure among the EMR [27] population.

Similarly, studies conducted in Saudi Arabia [7] and Bahrain [8] support the findings of the current study by reporting an increased prevalence of osteoporosis among men and postmenopausal women. In the Saudi Arabian study [7], the prevalence of osteoporosis among males increased from 24.3% in earlier reports to 28.2%, highlighting a rising trend. The same study also emphasized that osteoporosis in postmenopausal women remains a major public health concern. In Bahrain [8], 37.5% of postmenopausal women were found to have osteoporosis based on DEXA measurements, further confirming the heightened risk in this population group.

The present study shows a significant association between menopausal status and left hip *T*‐score, with a *p*‐value of 0.003. Women who have undergone menopause had a higher proportion of abnormal *T*‐scores compared to those who had not. This finding can be explained by the decline in estrogen levels during menopause, which accelerates bone loss through increased bone resorption, leading to decreased bone density and strength. This disruption in the bone remodeling process predisposes menopausal women to osteoporosis and fractures [28].

Additionally, factors such as age‐related decline in bone density, decreased physical activity, and hormonal changes contribute to the higher prevalence of abnormal T‐scores in menopausal women. Studies from various parts of the world [9, 10, 15] support this finding. Current study shows that individuals with a history of disc disorders exhibit a significantly higher proportion of abnormal *T* scores compared to those without such history. This finding aligns with previous research [29] indicating a link between intervertebral disc degeneration and osteoporosis, as both conditions may share common underlying mechanisms such as age‐related bone loss and alterations in bone microarchitecture. According to the current study, the prevalence of abnormal BMD was found to be more among males (56.8%) compared to females (45.6%). This is contrary to a study [30] conducted in Jiangsu (China) among 2711 participants, where 2.68% of males and 13.82% of females had osteoporosis. This may be attributed to the difference in environmental factors such as cultural differences in health screening as well as the sociodemographic characteristics of the two populations.

The current study found a significant association between the combination *T* score and age of the participants. The greatest proportion of abnormal BMD was found in the > 60 age category at 63.5%. This was consistent with the findings of a cohort study [31] performed in the rural areas of Henan province in China between the years 2015 and 2017. According to this study, the mean values of BMD declined significantly in both sexes with an increase in age. This finding can be attributed to multiple factors such as accelerated loss of bone tissue due to genetic factors and increased osteoclastic resorption of the bone tissue as well as environmental factors such as falls which can increase the risk of developing abnormal BMD [32].

There was a significant association observed between the combination *T* score and geographical origin in the current study, with the greatest abnormal *T* score belonging to the participants from the African region followed by the Eastern Mediterranean region. This is in contrast to a study [33] published in 2022 which aimed to estimate the disease burden of LBMD and its related fractures in 204 countries and territories from the year 1990–2019. According to this study, black people were found to have the lowest rates of fractures, with white females being 4.7 times more likely to getting a fragility fracture compared to black females and white males being 2.7 times more likely as compared to black males. This discrepancy between the two studies may be due to the unequal distribution of nationalities with higher distribution of participants belonging to the African region found in the current study. However, another study [34] indicates that despite the high levels of sunlight found in the Middle Eastern and African region, this region also has the highest levels of vitamin D deficiency. This hypovitaminosis D could be a contributing factor to osteoporosis.

The current study shows a significant association between the BMI of the participants and the combination *T* score, with 69.6% of the participants with underweight/normal BMI classification having an abnormal *T* score and 46.4% of the participants with obese/overweight BMI classification having an abnormal *T* score. According to a study [35] conducted to establish the relationship between obesity and osteoporosis, low body weight is an important risk factor in the development of LBMD and fractures. This is consistent with the results of this study and may be attributed to decreased bone strength, as well as, less soft tissue that may protect bone from forces, besides the increased risk of falls due to decreased strength of the muscles.

The findings of this study should be interpreted in light of several limitations. Firstly, the cross‐sectional design prevents establishing causal relationships between the identified factors and BMD outcomes. Secondly, the study population was not evenly distributed across regions, sex, and age, potentially introducing selection bias. Additionally, limited testing for vitamin D status and reliance on self‐reported data for lifestyle factors may have impacted the accuracy of the results. Finally, the generalizability of these findings to other populations is limited by regional and cultural differences in diet, lifestyle, and healthcare access. Future research involving larger, more diverse cohorts and longitudinal designs is recommended to validate and expand upon these findings.

## 5. Conclusion

Out of the 335 participants in this study, the majority were females aged 40–59 years and primarily from the African region, highlighting the demographic groups most represented in the analysis. The findings underscore the importance of considering sociodemographic factors in bone health assessments. Notably, the spine T‐score showed significant associations with nationality and menopausal status, suggesting that these factors play a critical role in BMD variations. Additionally, the majority of participants with osteopenia and osteoporotic ranges of BMD were from the Eastern Mediterranean region, emphasizing potential regional differences in bone health. The left hip T‐score was significantly associated with a history of disc disorders, indicating a possible link between musculoskeletal conditions and decreased bone density. This finding is particularly relevant for clinicians managing patients with disc disorders, as it underscores the need for comprehensive assessments to identify and address underlying BMD abnormalities. Furthermore, cross‐tabulation revealed significant associations between the spine *T*‐score and age, geographical origin, and menopausal status, while the combined *T*‐score showed significant links with sex, age, geographical origin, and BMI. These results emphasize the multifaceted nature of BMD determinants and highlights the need for tailored prevention and management strategies that account for these critical variables.

## Conflicts of Interest

The authors declare no conflicts of interest.

## Funding

No external funding was received for this study.

## Supporting Information

The original data sheet of the cross‐sectional record‐based research has been uploaded as the supporting information in the form of a single excel file.

## Supporting information


**Supporting Information** Additional supporting information can be found online in the Supporting Information section.

## Data Availability

The data that support the findings of this study are available in the supporting information of this article.
